# Composition and functional analysis of low-molecular-weight glutenin alleles with Aroona near-isogenic lines of bread wheat

**DOI:** 10.1186/1471-2229-12-243

**Published:** 2012-12-22

**Authors:** Xiaofei Zhang, Hui Jin, Yan Zhang, Dongcheng Liu, Genying Li, Xianchun Xia, Zhonghu He, Aimin Zhang

**Affiliations:** 1 Institute of Crop Science, National Wheat Improvement Center, Chinese Academy of Agricultural Sciences (CAAS), 12 Zhongguancun South Street, Beijing, 100081, China; 2 State Key Laboratory of Plant Cell and Chromosome Engineering, National Center for Plant Gene Research, Institute of Genetics and Developmental Biology, Chinese Academy of Sciences, 1 West Beichen Road, Beijing, 100101, China; 3 Crop Research Institute, Shandong Academy of Agricultural Sciences, Jinan, 250100, Shandong, China; 4 International Maize and Wheat Improvement Center (CIMMYT) China Office, c/o CAAS, 12 Zhongguancun South Street, Beijing, 100081, China

**Keywords:** Triticum aestivum, *Glu-3* alleles, Bread-making quality

## Abstract

**Background:**

Low-molecular-weight glutenin subunits (LMW-GS) strongly influence the bread-making quality of bread wheat. These proteins are encoded by a multi-gene family located at the *Glu-A3*, *Glu-B3* and *Glu-D3* loci on the short arms of homoeologous group 1 chromosomes, and show high allelic variation. To characterize the genetic and protein compositions of LMW-GS alleles, we investigated 16 Aroona near-isogenic lines (NILs) using SDS-PAGE, 2D-PAGE and the LMW-GS gene marker system. Moreover, the composition of glutenin macro-polymers, dough properties and pan bread quality parameters were determined for functional analysis of LMW-GS alleles in the NILs.

**Results:**

Using the LMW-GS gene marker system, 14–20 LMW-GS genes were identified in individual NILs. At the *Glu-A3* locus, two m-type and 2–4 i-type genes were identified and their allelic variants showed high polymorphisms in length and nucleotide sequences. The *Glu-A3d* allele possessed three active genes, the highest number among *Glu-A3* alleles. At the *Glu-B3* locus, 2–3 m-type and 1–3 s-type genes were identified from individual NILs. Based on the different compositions of s-type genes, *Glu-B3* alleles were divided into two groups, one containing *Glu-B3a*, *B3b*, *B3f* and *B3g*, and the other comprising *Glu-B3c*, *B3d*, *B3h* and *B3i*. Eight conserved genes were identified among *Glu-D3* alleles, except for *Glu-D3f*. The protein products of the unique active genes in each NIL were detected using protein electrophoresis. Among *Glu-3* alleles, the *Glu-A3e* genotype without i-type LMW-GS performed worst in almost all quality properties. *Glu-B3b, B3g* and *B3i* showed better quality parameters than the other *Glu-B3* alleles, whereas the *Glu-B3c* allele containing s-type genes with low expression levels had an inferior effect on bread-making quality. Due to the conserved genes at *Glu-D3* locus, *Glu-D3* alleles showed no significant differences in effects on all quality parameters.

**Conclusions:**

This work provided new insights into the composition and function of 18 LMW-GS alleles in bread wheat. The variation of i-type genes mainly contributed to the high diversity of *Glu-A3* alleles, and the differences among *Glu-B3* alleles were mainly derived from the high polymorphism of s-type genes. Among LMW-GS alleles, *Glu-A3e* and *Glu-B3c* represented inferior alleles for bread-making quality, whereas *Glu-A3d*, *Glu-B3b*, *Glu-B3g* and *Glu-B3i* were correlated with superior bread-making quality. *Glu-D3* alleles played minor roles in determining quality variation in bread wheat. Thus, LMW-GS alleles not only affect dough extensibility but greatly contribute to the dough resistance, glutenin macro-polymers and bread quality.

## Background

The unique viscoelastic properties conferred by gluten proteins in bread wheat are the basis of the flexible processing qualities in producing a wide range of food products for a large proportion of the world population. Gluten proteins, also named prolamins, are classically divided into gliadins and glutenins, based on different solubilities in an alcohol/water mixture [1]. The gliadins are generally monomeric proteins, divided into three groups, α/β-, γ- and ω-gliadins, based on their electrophoretic mobilities at low pH [2]. Glutenins form polymeric proteins stabilized by interchain disulfide bonds. Based on different molecular weights, glutenins can be classified into two groups, high-molecular-weight glutenin subunits (HMW-GS) and low-molecular-weight glutenin subunits (LMW-GS) [2,3]. LMW-GS are further divided into B-, C- and D-group subunits according to their mobilities in sodium dodecyl sulphate polyacrylamide-gel electrophoresis (SDS-PAGE) [4].

In bread wheat, HMW-GS are encoded by genes at the orthologous *Glu-1* loci on the long arms of chromosomes *1A*, *1B* and *1D* (*Glu-A1*, *Glu-B1* and *Glu-D1*). Each locus possesses two paralogous genes encoding one x- and one y-type subunit [5]. LMW-GS genes are located at the *Glu-A3*, *Glu-B3* and *Glu-D3* loci on the short arms of group 1 chromosomes. The LMW-GS genes at the *Glu-3* loci and the gliadin genes at the *Gli-1* loci are tightly linked and form gene clusters covering several centimorgans (cMs) [6-8]. Moreover, unlike the simple composition of HMW-GS, LMW-GS are encoded by a complex multigene family without the information of the exact number of genes [9,10]. A large number of genes and abundant allelic variations at *Glu-3* loci and their tight linkage with gliadin genes make it difficult to elucidate the composition and function of LMW-GS genes in bread wheat [4].

SDS-PAGE is widely used to investigate the abundant seed storage proteins in bread wheat. Based on the mobility of proteins in SDS-PAGE gels, whole seed proteins are divided into four groups, HMW-GS, D-group, B-group, and C-group. D-group proteins are mainly composed of ω-gliadin proteins, whereas B-group mostly consists of LMW-GS proteins, and C-group comprise α, β and γ-type gliadins and several LMW-GS proteins [4]. Based on the different electrophoretic patterns, LMW-GS protein alleles encoded by *Glu-3* loci are designated alphabetically (e.g., *Glu-A3a*) [11]. However, identification of the LMW-GS composition in breeding programs remains a significant challenge because determination of LMW-GS alleles with SDS-PAGE needs much experience. This is why the functions of LMW-GS alleles are not well characterized. Gene-specific markers for *Glu-A3* and *Glu-B3* alleles were developed to identify different LMW-GS alleles. However, molecular markers for *Glu-D3* alleles are still not available due to the slight differences among alleles [12-17]. Using BAC library screening and proteomics methods, LMW-GS genes in Norin 61 (*Glu-A3d*, *Glu-B3i* and *Glu-D3c*), Glenlea (*Glu-A3g*, *Glu-B3g* and *Glu-D3c*) and Xiaoyan 54 (*Glu-A3d*, *Glu-B3d* and *Glu-D3c*) were identified and characterized [10,18-20]. These studies greatly improved our understanding of the unique genes encoding different LMW-GS alleles in bread wheat. Recently, based on the conserved and polymorphic structure of LMW-GS genes, we developed a LMW-GS gene marker system and a full-length gene cloning method [21,22]. They were successfully used to identify and characterize more than 16 LMW-GS genes in individual wheat varieties [21]. Both methods are helpful in elucidating the composition of *Glu-3* alleles in LMW-GS genes of bread wheat.

The effects of glutenin alleles on dough properties and processing qualities were mostly studied in two types of populations: structured populations (e.g., recombinant inbred lines (RILs) and doubled haploid lines) derived from biparental crosses, and non-structured populations, general collections of varieties and breeding lines. Due to the simple composition and easy identification of allelic variants of HMW-GS, the contributions of HMW-GS to dough properties and end-use quality were well investigated and widely used in breeding programs [23]. However, HMW-GS alone could not explain the variation in quality among wheat varieties, as LMW-GS also contributed to dough properties [24-31]. For example, LMW-GS alleles made a slightly larger contribution than HMW-GS to dough extensibility [32,33]. Compared with HMW-GS, LMW-GS formed highly polymorphic protein complex and contain abundant allelic variation. Using SDS-PAGE and allele-specific primers, LMW-GS alleles were identified in wheat collections or structured populations, and their effects on processing quality were analyzed and discussed. However, controversies were common in regard to different kinds of populations or collections. For example, Cane et al. [34] and Eagles et al. [33] reported that *Glu-A3e* was correlated with inferior dough resistance and extensibility, whereas Zheng et al. [35] showed that *Glu-A3e* was a favorable allele for dough-mixing properties. Due to the complex composition of LMW-GS alleles and difficulties in distinguishing LMW-GS from gliadins in SDS-PAGE gels, the molecular genetic mechanisms behind the functional differences of LMW-GS alleles are not well investigated.

In the present study, a set of near-isogenic lines (NILs) containing five *Glu-A3* alleles, eight *Glu-B3* alleles and five *Glu-D3* alleles, was used to study the effects of LMW-GS on the composition of glutenin macro-polymers (GMP), dough properties, and pan bread making quality. These NILs were investigated using SDS-PAGE, the LMW-GS gene marker system and two-dimensional gel electrophoresis (2D-PAGE) for identifying the composition of LMW-GS genes and proteins in each LMW-GS allele, and analyzing their association with dough properties and bread-making quality.

## Results

### Separation of LMW-GS proteins in Aroona NILs using SDS-PAGE

**Figure 1:**
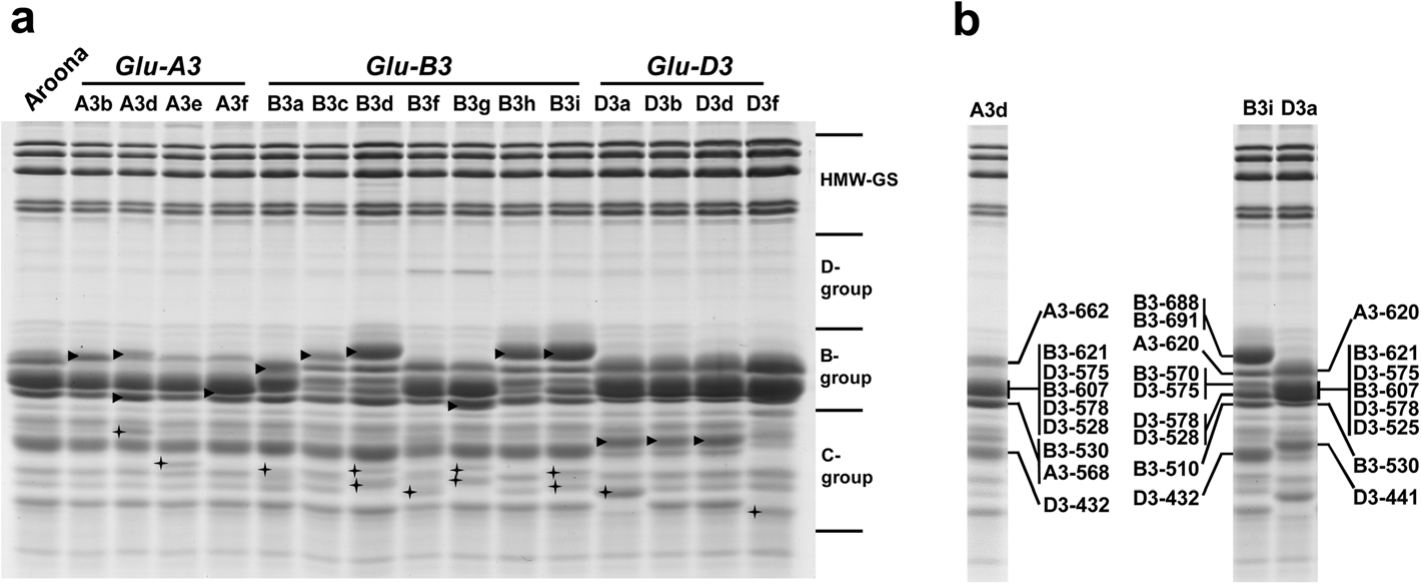
**a) Separation of glutenin proteins from flour of Aroona NILs.** Arrowheads mark unique LMW-GS protein bands in individual NILs, and asterisks label specific gliadin bands. **b**) Identification of LMW-GS bands in Aroona-*Glu-A3d*, Aroona-*Glu-B3i* and Aroona-*Glu-D3a.*

The glutenin alleles in the flour of 16 Aroona NILs were separated by SDS-PAGE (Figure 1a). Aroona possessed five HMW-GS proteins, viz., 1, 7 + 9 and 2 + 12, encoded by genes at *Glu-A1*, *Glu-B1* and *Glu-D1* loci, respectively. All NILs had the same HMW-GS as Aroona, and their unique LMW-GS and gliadin bands were labeled in Figure 1a. Four *Glu-A3* NILs possessed unique LMW-GS bands at the B-group region from Aroona (*Glu-A3c*). Aroona-*Glu-A3d* and Aroona-*Glu-A3e* also contained specific gliadin bands (Figure 1a). Among eight *Glu-B3* NILs, Aroona (*Glu-B3b*), Aroona-*Glu-B3a*, *B3f* and *B3g* shared similar B-group proteins, whereas Aroona-*Glu-B3c*, *B3d*, *B3h* and *B3i* showed another group of electrophoretic patterns (Figure 1a). Among the latter four NILs, Aroona-*Glu-B3c* possessed the lowest quantity of B-group LMW-GS proteins (Additional file 1: Figure S1), especially the protein with the largest molecular weight. Four *Glu-D3* NILs and Aroona shared the same B-group LMW-GS proteins (Figure 1a). Aroona-*Glu-D3b* and *D3d* had the same protein bands and contained one unique LMW-GS from Aroona. Except for one gliadin protein, the electrophoretic pattern of Aroona-*Glu-D3a* was the same as those of Aroona-*Glu-D3b* and *D3d*. Aroona-*Glu-D3f* produced a quite different protein pattern in the C-group region compared with other *Glu-D3* NILs. Generally, the *Glu-3* loci were tightly linked with *Gli-1* loci [6-8], and it was difficult to break this linkage through genetic recombination in conventional crosses. Thus, each *Glu-3* NIL not only possessed unique LMW-GS but also contained 1 or 2 specific gliadins.

## Dissecting genes encoding LMW-GS alleles in the Aroona NILs

**Figure 2:**
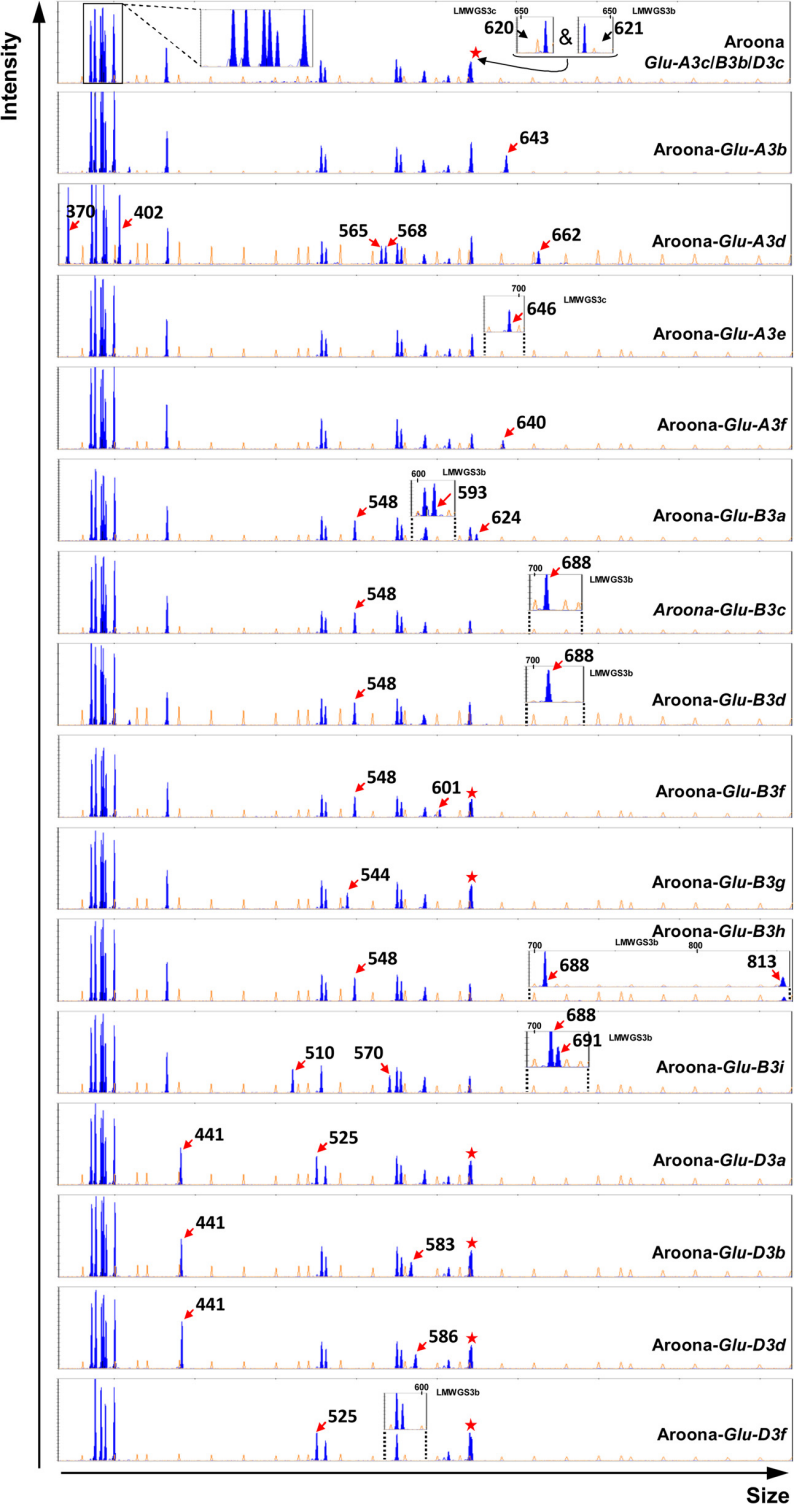
**Separation of LMW-GS genes in Aroona NILs using the LMW-GS gene molecular marker system.** The marker system contained three sets of primers, LMWGS1, LMW-GS2 and LMWGS3. The main data were derived from primer set LMWGS1, and the added figures were from the primer LMWGS3b or LMWGS3c. The peaks marked with the asterisk comprise two genes, *A3-620* and *B3-621*. The arrows indicate unique genes in each Aroona NIL.

To analyze the genes encoding LMW-GS alleles in Aroona NILs, we investigated the 16 Aroona NILs using a previously developed LMW-GS gene marker system, that was efficient in separating LMW-GS genes in bread wheat (Figure 2; Table 1) [21,22]. Eighteen LMW-GS genes were identified in Aroona, including four *Glu-A3* genes (*A3-391*, *A3-400*, *A3-502-2* and *A3-620*), four *Glu-B3* genes (*B3-530-2*, *B3-578*, *B3-607* and *B3-621-1*), and eight *Glu-D3* genes (*D3-385*, *D3-393*, *D3-394*, *D3-432*, *D3-528*, *D3-575*, *D3-578-1* and *D3-591*) (Table 1). The other two novel genes corresponding to DNA fragments 388 and 410 present in all 16 lines (Table 1) were pseudogenes with premature termination codons in the CDS regions. Among 16 Aroona NILs, Aroona-*Glu-D3f* possessed 14 unique genes, with the least number of LMW-GS genes, whereas Aroona-*Glu-A3d* had the largest number of LMW-GS genes (20 genes). Comparison of the LMW-GS genes among NILs indicated that each NIL differed from Aroona at only one *Glu-3* locus. With the help of the complete gene sequences available in Aroona NILs and some other wheat varieties [10,13,14,17-19,21], the composition of LMW-GS genes in each Aroona NIL was well characterized.

**Table 1:** LMW-GS genes identified in 16 Aroona NILs using the LMW-GS gene molecular marker system

**Line**	**Genes at *Glu-A3 *locus**						**Genes at *Glu-B3 *locus**							**Genes at *Glu-D3 *locus**								**New genes**	
Aroona(*Glu-A3c, B3b, D3c*)	391	400	502-2			**620**^**a**^	**530-2**			578	**607**	**621-1**		**385**	393	**394**	**432**	**528**	**575**	**578-1**	591	388	410
Aroona-*Glu-A3b*	391	400	502-1			**643**	**530-2**			578	**607**	**621-1**		**385**	393	**394**	**432**	**528**	**575**	**578-1**	591	388	410
Aroona-*Glu-A3d*	370	**402**	484	565	**568**	**662**	**530-2**			578	**607**	**621-1**		**385**	393	**394**	**432**	**528**	**575**	**578-1**	591	388	410
Aroona-*Glu-A3e*	391	400	502-3			**646**	**530-2**			578	**607**	**621-1**		**385**	393	**394**	**432**	**528**	**575**	**578-1**	591	388	410
Aroona-*Glu-A3f*	391	400	502-4		**573**	640	**530-2**			578	**607**	**621-1**		**385**	393	**394**	**432**	**528**	**575**	**578-1**	591	388	410
Aroona-*Glu-B3a*	391	400	502-2			**620**	**530-1**	548		578	**593**	**624**		**385**	393	**394**	**432**	**528**	**575**	**578-1**	591	388	410
Aroona-*Glu-B3c*	391	400	502-2			**620**	**530-1**	548				**688-1**		**385**	393	**394**	**432**	**528**	**575**	**578-1**	591	388	410
Aroona-*Glu-B3d*	391	400	502-2			**620**	**530-1**	548				**688-2**		**385**	393	**394**	**432**	**528**	**575**	**578-1**	591	388	410
Aroona-*Glu-B3f*	391	400	502-2			**620**	**530-3**	548		578	**601**	**621-1**		**385**	393	**394**	**432**	**528**	**575**	**578-1**	591	388	410
Aroona-*Glu-B3g*	391	400	502-2			**620**	**530-3**			578	**544**	**621-2**		**385**	393	**394**	**432**	**528**	**575**	**578-1**	591	388	410
Aroona-*Glu-B3h*	391	400	502-2			**620**	**530-2**	548				**688-3**	813	**385**	393	**394**	**432**	**528**	**575**	**578-1**	591	388	410
Aroona-*Glu-B3i*	391	400	502-2			**620**	**510**		**570**			**688-4**	**691**	**385**	393	**394**	**432**	**528**	**575**	**578-1**	591	388	410
Aroona-*Glu-D3a*	391	400	502-2			**620**	**530-2**			578	**607**	**621-1**		**385**	393	**394**	**441**	**525**	**575**	**578-2**	591	388	410
Aroona-*Glu-D3b*	391	400	502-2			**620**	**530-2**			578	**607**	**621-1**		**385**	393	**394**	**441**	**528**	**575**	**578-2**	583	388	410
Aroona-*Glu-D3d*	391	400	502-2			**620**	**530-2**			578	**607**	**621-1**		**385**	393	**394**	**441**	**528**	**575**	**578-2**	586	388	410
Aroona-*Glu-D3f*	391	400	502-2			**620**	**530-2**			578	**607**	**621-1**		--	--	**394**	--	**525**	**575**	**578**	--	388	410

^**a**^ Bold numbers indicate active LMW-GS genes.

For *Glu-A3* alleles, 4–6 genes were identified in Aroona (*Glu-A3c*) and four *Glu-A3* NILs (Table 1; Figure 2). Except for Aroona-*Glu-A3d*, these NILs possessed LMW-GS genes *A3-391* and *A3-400* (Table 1; Figure 2), which were m-type pseudogenes with premature termination codons. The other genes were i-type genes. The *A3-502* gene contained four allelic variants with unique SNPs and InDels, i.e., *A3-502-1*, *A3-502-2*, *A3-502-3* and *A3-502-4*. All *A3-502* allelic variants and the *A3-640* gene were pseudogenes, containing premature termination codons in the coding sequences. The i-type genes, *A3-643*, *A3-620*, *A3-646* and *A3-573*, were the only active genes in Aroona-*Glu-A3b*, *A3c*, *A3e* and *A3f*, respectively. Sequence alignments showed that *A3-620* and *A3-643* shared high identity (> 99%) differing only in one InDel and one SNP, whereas *A3-573* and *A3-646* were greatly different from each other and the other i-type genes (Additional file 1: Figure S2). A3-573 was much shorter than the other i-type proteins due to deletion of three repetitive units and several glutamine residues in repetitive regions, and lacking GTFLQPH in the C-terminal domain (Additional file 1: Figure S2). Aroona-*Glu-A3d* contained six unique LMW-GS genes (Table 1; Figure 2). For two m-type genes, *A3-370* was a pseudogene, whereas *A3-402* contained an intact coding sequence that could be expressed in the developing grain [18]. The other four genes, *A3-484*, *A3-565*, *A3-568* and *A3-662* were i-type genes, and among them *A3-568* and *A3-662* were active. Both genes contained unique SNPs in the C-terminal domain and different lengths of repetitive region compared to other i-type genes (Additional file 1: Figure S2). Thus, compared to only one LMW-GS gene expressed in the other *Glu-A3* alleles, *Glu-A3d* contained three active genes, one m-type (*A3-402*) and two i-type (*A3-568* and *A3-662*) LMW-GS genes. Except for *A3-402*, the active genes at the *Glu-A3* locus were i-type genes. These i-type genes encoded B-group LMW-GS, producing different electrophoretic patterns in the B-group region among *Glu-A3* NILs (Figure 1).

For *Glu-B3* alleles, 3–5 LMW-GS genes were identified in Aroona (*Glu-B3b*) and seven *Glu-B3* NILs. Gene *B3-530* was present in all Aroona NILs and four allelic variants (*B3-530-1*, *-2*, *-3* and *B3-510*) were identified (Table 1). Sequence alignment showed that the deduced protein sequences of the three *B3-530* allelic variants differed in only four amino acids, whereas B3-510 contained a deletion in the repetitive domain and differed by seven amino acids from the B3-530 proteins (Additional file 1: Figure S3). *B3-544*, *B3-593*, *B3-601* and *B3-607* were allelic variants, sharing over 99% of identity and their deduced protein sequences differed only in the repetitive domain with the deletion of glutamine residues or one repetitive unit (Additional file 1: Figure S4). *B3-621-1*, *B3-621-2* and *B3-624* genes were highly conserved and their proteins differed only in one InDel of a glutamine residue in the repetitive domain and one amino acid at the C terminal region (Additional file 1: Figure S5). The *B3-688* gene was quite distinct, differing from *B3-621* and *B3-624* in several SNPs and InDels (Additional file 1: Figure S5). Moreover, *B3-688* varied among Aroona*-Glu-B3c*, *B3d*, *B3h* and *B3i*, and the protein products differed in seven amino acids and two InDels (Additional file 1: Figure S5). Among *Glu-B3* genes, *B3-548*, *B3-578* and *B3-813* were pseudogenes with premature termination codons, whereas the other genes contained intact ORFs (Open Reading Frame). Sequence analysis showed that *B3-530*, *B3-510*, *B3-548* and *B3-570* were m-type genes and the others were s-type genes. Based on the differences in s-type genes, *Glu-B3* NILs were classified into two groups. Aroona-*Glu-B3a*, *B3b*, *B3f* and *B3g* contained *B3-578*, *B3-544*/*593*/*601*/*607*, and *B3-621*/*624*, and the others had longer sequences of genes, *B3-68*8 and *B3-691*/*813*/*Null* (Table 1). This classification was consistent with the different electrophoretic patterns contrasting the two groups (Figure 1a).

Aroona-*Glu-B3a*, *B3b*, *B3f* and *B3g* had similar LMW-GS compositions, but each of them possessed unique allelic variants (Table 1; Figure 2). Both Aroona-*Glu-B3f* and *B3g* contained B3-530-3, but they differed in *B3-544*, *B3-601*, *B3-621-1* and *B3-621*-*2* genes (Table 1; Additional file 1: Figures S4 and S5).Aroona-*Glu-B3f* and Aroona (*Glu-B3b*) shared the same *B3-621-1*, but possessed different genes *B3-601*/*B3-530-3* and *B3-607*/*B3-530-2*, respectively (Table 1; Additional file 1: Figures S3 and S4). On the other hand, Aroona-*Glu-B3c*, *B3d*, *B3h*, and *B3i* also differed in active genes and their allelic variants. Aroona-*Glu-B3c* and *B3d* contained B3-530-1, but their B3-688 proteins differed in five amino acids (Table 1; Additional file 1: Figure S5). Aroona-*Glu-B3i* was unique, containing four active LMW-GS genes *B3-510*, *B3-570*, *B3-688-4* and *B3-691* (Table 1; Figure 2). *B3-570* was only present in *Glu-B3i*, encoding an m-type LMW-GS with the N-terminal domain METSQIPGLEKPS. Although *B3-688-4* and *B3-691* were different genes, they shared about 99% identity and *B3-691* is formally reported for the first time.

At the *Glu-D3* locus, eight genes were identified from individual NILs except Aroona-*Glu-D3f* (Table 1). *D3-393* and *D3-583*/*586*/*591* had premature termination codons. All the other genes were widely reported and were expressed in developing grains [10,17,18,20]. *D3-385*, *D3-394* and *D3-575* were highly conversed among the Aroona NILs. *D3-432* and *D3-441* were allelic variants with over 99% identity, and differed in only two SNPs and two InDels. Allelic variants *D3-525* and *D3-528* were identical, except for one InDel. Two allelic variants, *D3-578-1* and *D3-578-2* differed by eight SNPs (Table 1). Compared with the active *Glu-D3* genes in Aroona (*Glu-D3c*), *D3-441* and *D3-578-2* were present in Aroona-*Glu-D3b* and *D3d*, which possessed the same active LMW-GS genes (Table 1; Figure 2). Besides *D3-441* and *D3-578-2*, Aroona-*Glu-D3a* contained unique gene *D3-525* in contrast to Aroona. In Aroona-*Glu-D3f*, only four LMW-GS genes were identified; the other four genes might be absent due to deletion, substitution or other mutations (Table 1; Figure 2).

Each Aroona NIL possessed unique LMW-GS alleles. The gene compositions of these LMW-GS alleles were dissected, all the LMW-GS gene sequences were characterized, and the proteins sequences were deduced (Table 1; Figure 2). Based on the size of the deduced LMW-GS and the LMW-GS 2D-PAGE spots identified from Xiaoyan 54, Jing 411 and Norin 61 [18,19], LMW-GS genes were assigned to protein bands in SDS-PAGE gels (Figure 1b). The active i-type genes at the *Glu-A3* locus, all the active genes at *Glu-B3* and the genes *D3-525*/*528*, *D3-575* and *D3-578* at *Glu-D3* encoded B-group LMW-GS, whereas the protein products of the other active genes belonged to the C-group. Moreover, *B3-621/624*, *B3-593/601/607*, *D3-575* and *D3-578* encoded proteins with similar molecular weights (38–40 kDa), and ran synchronously on SDS-PAGE, forming the thickest protein bands in Aroona-*Glu-B3a*, *B3b* and *B3f*. *B3-688* genes encoding the longest protein conferred the unique protein pattern on Aroona-*GluB3d*, *B3h* and *B3i*. (Figure 1).

## Comparison of whole proteins among LMW-GS NILs using 2D-PAGE

**Figure 3:**
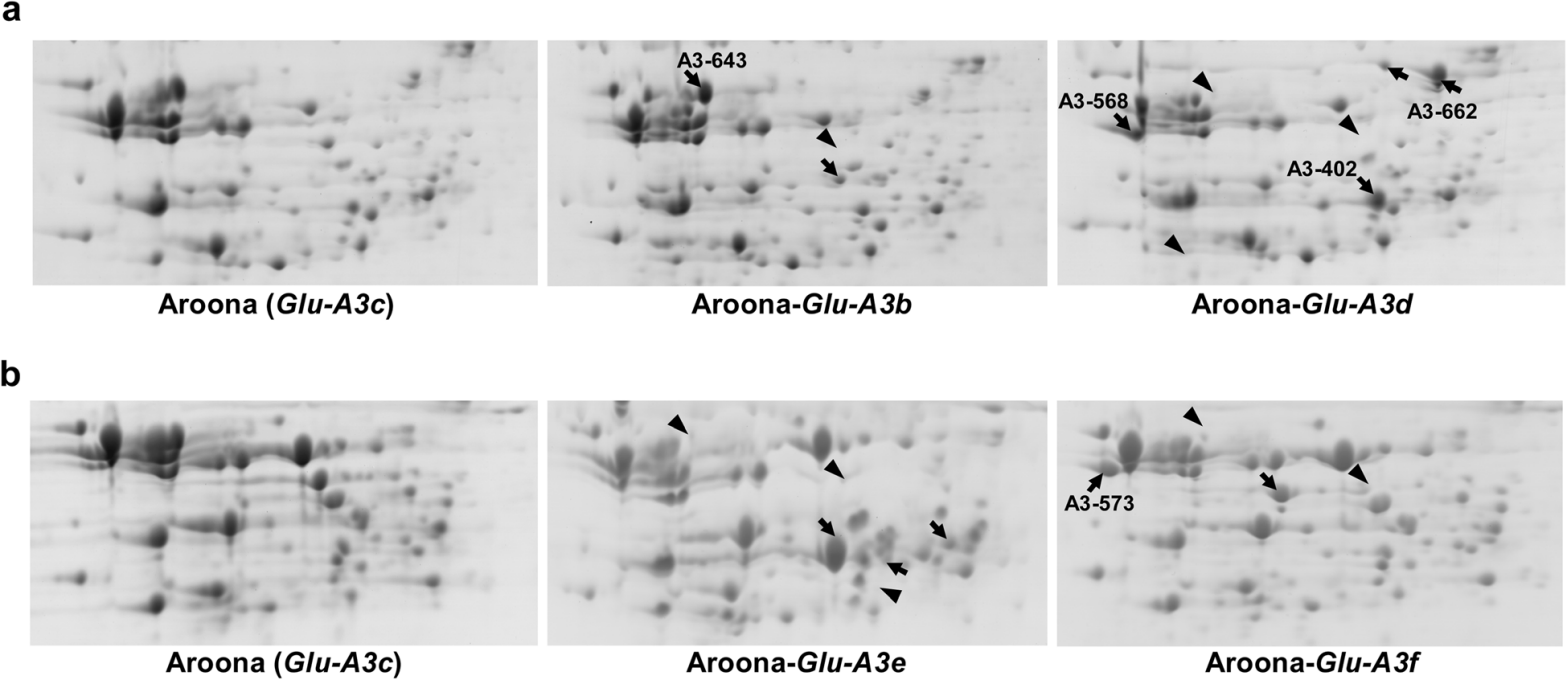
**Separation of flour proteins in *Glu-A3 *NILs using two-dimensional gel electrophoresis (2D-PAGE). a**) Comparison of storage proteins from Aroona, Aroona-*Glu-A3b* and Aroona-*Glu-A3d*. **b**) Comparison of storage proteins from Aroona, Aroona-*Glu-A3e* and Aroona-*Glu-A3f*. The arrows indicate the unique protein spots in each NIL, and the arrowheads show the protein spots present in Aroona but absent in other NILs. The high molecular weight glutenin subunit protein spots are the same and not shown here due to limited space.

**Figure 4:**
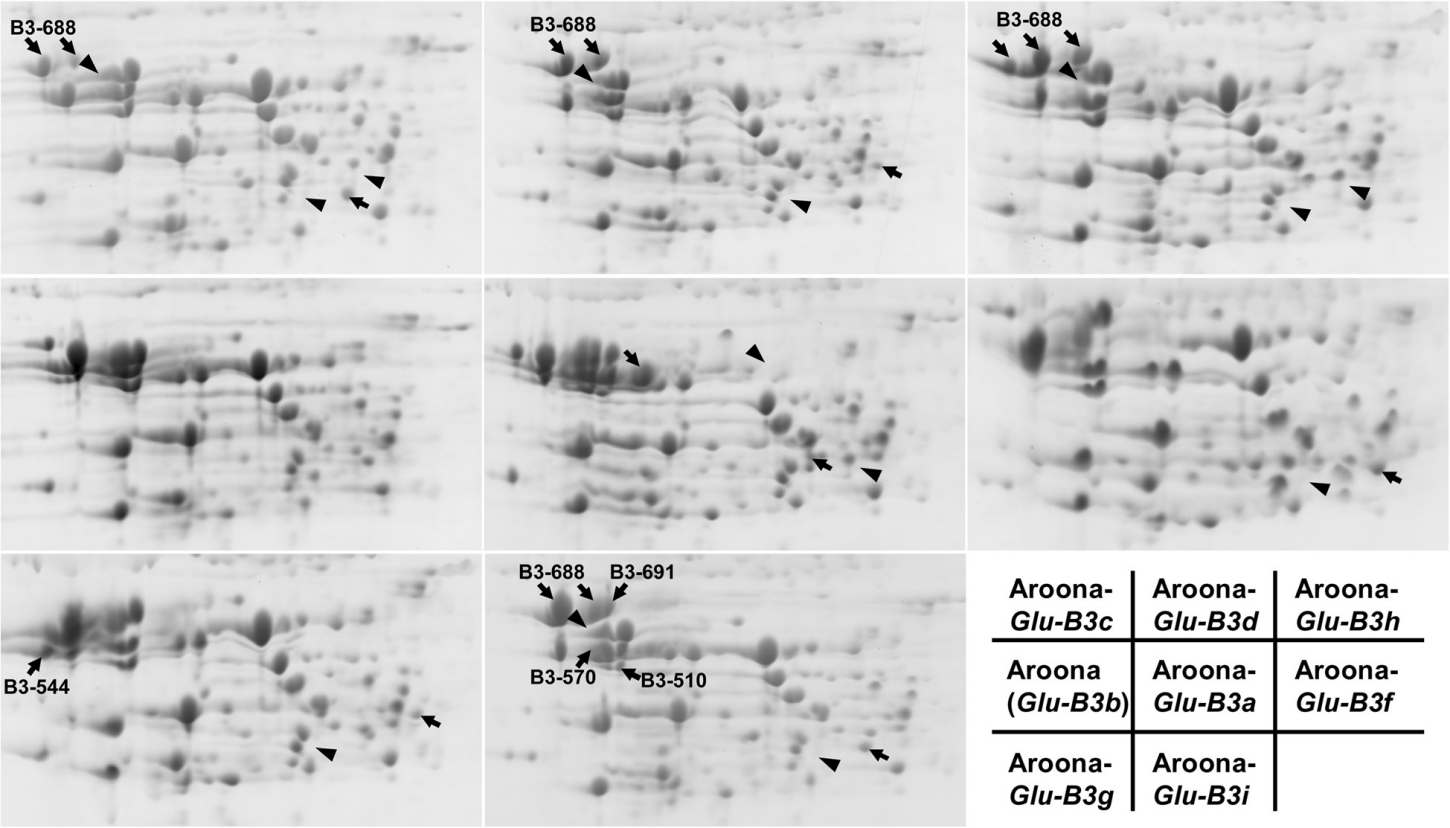
**Separation of flour proteins in eight *Glu-B3 *NILs using two-dimensional gel electrophoresis (2D-PAGE).** Arrows indicate the unique protein spots in each NIL, and the arrowheads show protein spots present in Aroona but absent in other NILs.

**Figure 5:**
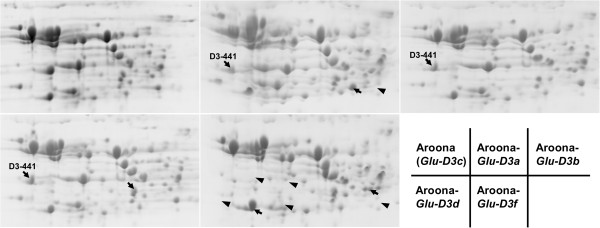
**Separation of flour proteins in five *Glu-D3 *NILs using two-dimensional gel electrophoresis (2D-PAGE).** The arrows indicate the unique protein spots in each NIL, and the arrowheads show the protein spots present in Aroona but absent in other NILs.

The data from SDS-PAGE and the LMW-GS marker system showed that each *Glu-3* allele contained several unique LMW-GS and gliadin genes. To analyze the composition of LMW-GS and gliadin proteins in individual NILs, we performed 2D-PAGE analysis of the whole flour proteins (Figures 3, 4 and 5). In the previous studies, using 2D-PAGE coupled with MS or N-terminal sequencing technology, LMW-GS proteins in Xiaoyan 54, Jing 411, Norin 61 and Butte 86 were successfully identified [18,19,36]. These data greatly contributed to the identification of flour proteins in the Aroona NILs.

Among five *Glu-A3* NILs, Aroona-*Glu-A3b* and Aroona (*Glu-A3c*) shared the same protein patterns, except for one gliadin spot, which was in agreement with the similar composition of LMW-GS genes and the high identity between *A3-620* and *A3-643* (Figure 3a). Compared with Aroona, Aroona-*Glu-A3d* contained three unique LMW-GS, A3-402, A3-568 and A3-662, and two gliadin spots were absent (Figure 3a). For Aroona-*Glu-A3e*, the protein product of the unique i-type gene *A3-646* was too little to be detected, but three unique gliadin spots were present in 2D-PAGE (Figure 3b). Aroona-*Glu-A3f* also contained one unique i-type protein (A3-573) and one gliadin spot different from Aroona (Figure 3b). Thus, accompanying the unique LMW-GSs in individual *Glu-A3* NILs, generally 1–2 gliadin spots were different among them. The only exception was Aroona-*Glu-A3e* which possessed the highest number of gliadins and the lowest quantity (none) of i-type LMW-GS among the *Glu-A3* NILs (Figure 3b).

Among eight *Glu-B3* NILs, Aroona-*Glu-B3c*, *B3d*, *B3h* and *B3i* shared similar protein spot patterns on 2D-PAGE, consistent with their similar electrophoretic patterns on SDS-PAGE (Figures 1a and 4). Compared with the protein spot pattern of Aroona (*Glu-B3b*), Aroona-*Glu-B3c*, *B3d*, *B3h* and *B3i* contain the unique B3-688 proteins (Figure 4). The B3-688 protein spot in Aroona-*Glu-B3c* was much weaker than those in the other three NILs (Additional file 1: Figure S6). Except for LMW-GS proteins, only one or two small gliadin spot differences were identified among the four NILs (Figure 4). The other three Aroona NILs (i.e., Aroona-*Glu-B3a*, *B3f* and *B3g*) had similar protein spot patterns with Aroona (*Glu-B3b*; Figure 4). Among these four NILs, a unique LMW-GS protein B3-544 was detected in Aroona-*Glu-B3g*, whereas the other three alleles shared the same LMW-GS protein spot patterns (Figure 4). Thus, the 2D-PAGE patterns of Aroona and seven *Glu-B3* NILs suggested that their different proteins mainly were unique LMW-GS proteins rather than gliadins.

For the *Glu-D3* NILs, the protein spots encoded by the *D3-441* gene in Aroona-*Glu-D3a*, *D3b* and *D3d* had larger molecular weights and pIs than its allelic variant D3-432 in Aroona (Figure 5). No other different LMW-GS spots were detected among these *Glu-D3* NILs, and Aroona-*Glu-D3a* and *D3d*, each contained only one unique gliadin spot (Figure 5). The Aroona-*Glu-D3f* allele was unique, lacking two LMW-GS proteins (i.e., D3-385 and D3-441) and at least three medium gliadin spots (Figure 5). The absence of four LMW-GS genes was also observed using the LMW-GS gene marker system (Table 1). Thus, except for Aroona-*Glu-D3f*, only one or two different protein spots were detected among *Glu-D3* NILs, and the proteins encoded by genes at the *Glu-D3* locus were highly conserved.

## Quality properties of the LMW-GS NILs

Dough properties such as Zeleny-sedimentation value (ZSV), Farinograph and Extensograph parameters, GMP parameters, and pan bread quality parameters, were measured on the Aroona NILs (Table 2). The 16 genotypes showed significant differences (*P* < 0.05 or *P* < 0.01) in most quality parameters, suggesting that genotype had an important influence on variation in wheat quality properties (Additional file 2: Table S1). Significant differences in some parameters (e.g., kernel protein, ZSV, Farinograph water absorption, glutenin and gliadin contents, and external bread color and structure) were also observed between the two locations (Additional file 2: Table S1). However, no significant interaction effects between genotypes and locations were detected for most quality parameters, except for external color of bread and Farinograph development time (Additional file 2: Table S1). Analysis of variance (ANOVA) and multiple comparisons of all quality parameters were performed among all NILs within each *Glu-3* group (Table 2, Additional file 2: Tables S2, S3 and S4).

**Table 2:** **F values of one way ANOVA of wheat quality parameters within each *Glu-3 *group**^**a**^

**Group**	**LV**	**LVS**	**EC**	**Sha**	**IC**	**Smo**	**Spr**	**Str**	**TF**	**PBTS**
Aroona-*Glu-A3*	5.31*	2.33	0.7	3.35	1.14	12.14**	0.67	6.45*	2	4.75*
Aroona-*Glu-B3*	2.85*	2.07	0.8	1.57	1.28	2.09	0.75	5.09**	2	5.78**
Aroona-*Glu-D3*	3.74	2.29	1.71	2.57	1	1	0.33	0.52	0.12	0.68
**Group**	**KP**	**ZSV**	**ST**	**DT**	**Wab**	**Rmax**	**EA**	**Ext**		
Aroona-*Glu-A3*	3.3	59.35**	6.55*	11.57**	27.98**	10.34**	13.46**	4.51*		
Aroona-*Glu-B3*	2.54	43.89**	6.19**	8.47**	5.4**	9.97**	10.06**	2.34		
Aroona-*Glu-D3*	3.01	0.85	2.47	3.05	4.04*	2.58	1.18	2.33		
**Group**	**Glutenin**	**Gliadin**	**Gli/Glu**	**EPP**	**UPP**	**%UPP**				
Aroona-*Glu-A3*	0.28	3.13	4.62*	2.87	7.1**	9.58**				
Aroona-*Glu-B3*	0.55	8.81**	4.65**	2.14	3.15*	3.63*				
Aroona-*Glu-D3*	3.64	0.32	6.55*	1.51	1.53	1.48				

* Significant at *P* < 0.05; ** Significant at *P* < 0.01.

^a^ LV: Loaf volume (cm^3^), LVS: Loaf volume score, EC: External color, Sha: Shape, IC: Inner color, Smo: Smoothness, Spr: Springiness, Str: Structure, TF: Taste flavor, PBTS: Pan bread total score, KP: Kernel protein (%, 14% m.b.), ZSV: Zeleny sedimentation value (mL), ST: Farinograph stability time (min), DT: Farinograph development time (min), Wab: Farinograph water absorption (%), Rmax: Extensograph maximum resistance (B.U.), EA: Energy area (cm^2^), Ext: Extensograph extensibility (mm), EPP: Extractable glutenin polymeric protein; UPP: Unextractable glutenin polymeric protein; Gli/Glu: Ratio of gliadin to glutenin; %UPP = UPP/(UPP + EPP) × 100.

**Figure 6:**
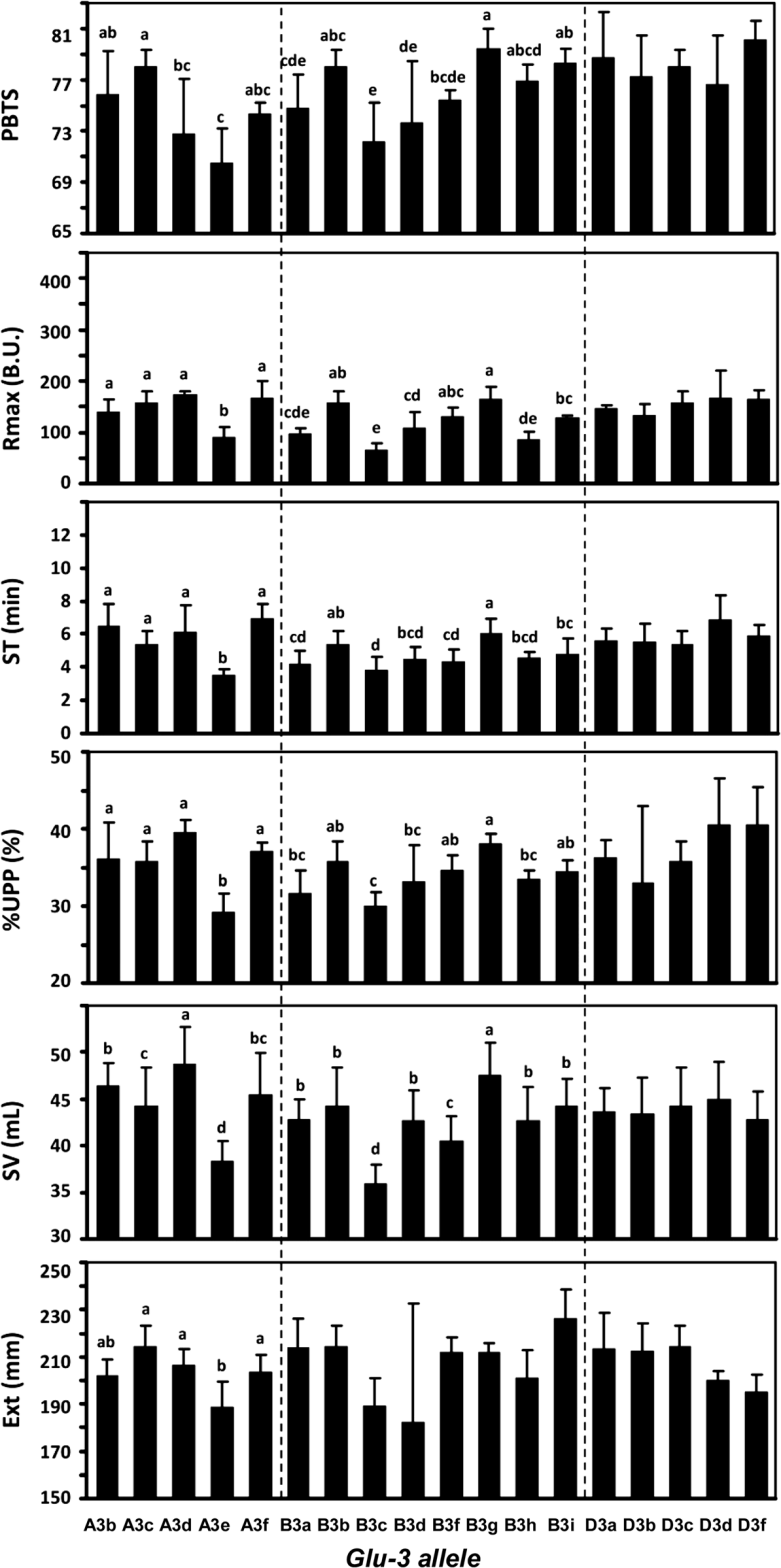
**Comparison of wheat quality properties among *Glu-A 3*NILs, *Glu-B3 *NILs and *Glu-D3 *NILs.** Statistical analysis of the quality parameters of Aroona NILs for each glutenin locus by ANOVA and Duncan LSR (*P* < 0.05). The bars are labeled by different letters or letter combinations based on multiple statistical comparisons. No statistical significance exists between alleles labeled by one or more identical letters. PBTS, Pan bread total score; Rmax, Extensograph maximum resistance; ST, Farinograph stability time; %UPP, Percent of SDS-unextractable fraction in total polymeric protein; ZSV, Zeleny-sedimentation value; Ext, Extensograph extensibility.

ANOVA showed that the *Glu-A3* NILs were significantly different in most quality parameters (Table 2). This suggested that *Glu-A3* alleles affected the bread-making quality of bread wheat. Subsequent multiple comparisons indicated that Aroona-*Glu-A3e* always produced the worst quality parameters, e.g., ZSV, Farinograph stability time (ST), Extensograph maximum resistance (Rmax), Extensograph extensibility (Ext), percentage of SDS-unextractable fraction in total polymeric protein (%UPP), and pan bread total score (PBTS), whereas the other five *Glu-A3* NILs had similar values for Rmax, ST, %UPP and Ext (Figure 6). Although Aroona-*Glu-A3d* had the largest ZSV, it showed only moderate PBTS (Figure 6).

*Glu-B3* NILs showed large differences in the most important quality parameters (Table 2), indicating that *Glu-B3* alleles were also significantly associated with bread-making quality. Among eight *Glu-B3* NILs, Aroona-*Glu-B3g* produced the highest PBTS, ZSV, ST, Rmax, %UPP and some other parameters (Figure 6, Additional file 2: Tables S2, S3 and S4). Similarly, Aroona (*Glu-B3b*) and Aroona-*Glu-B3i* had higher quality parameters than the other *Glu-B3* NILs, excluding Aroona-*Glu-B3g*. In contrast, Aroona-*Glu-B3c* performed the lowest in ZSV, ST, Rmax, %UPP, PBTS, and some other parameters (Figure 6; Additional file 2: Tables S2, S3 and S4), suggesting that *Glu-B3c* was an undesirable allele for bread-making quality. The other *Glu-B3* NILs (Aroona-*Glu-B3a*, *B3d*, *B3f* and *B3h*) possessed similar moderate quality parameters, without significant differences.

Five *Glu-D3* NILs produced similar quality parameters, without significant differences, except for Farinograph water absorption and the ratio of gliadin to glutenin (Table 2). This was consistent with the similar composition of LMW-GS genes and proteins among *Glu-D3* NILs.

## Discussion

In the present study, SDS-PAGE, 2D-PAGE and LMW-GS gene molecular marker system were used for characterization of LMW-GS genes and proteins in 16 Aroona NILs. Each NIL contained a unique LMW-GS allele. The genetic and protein compositions of each LMW-GS allele were dissected. In addition, we analyzed the functional differences among the LMW-GS alleles in bread-making quality using these NILs. The molecular mechanisms behind the functional differences were discussed based on the characterization of LMW-GS genes and proteins in the NILs.

## Characterization of LMW-GS genes and proteins in the NILs

SDS-PAGE is a useful method to accurately identify HMW-GS in bread wheat, but is inefficient for separating LMW-GS alleles, because of the presence of large numbers of LMW-GS proteins with similar mobilities with each other and with gliadins (Figure 1b). In the present study, the LMW-GS gene marker system was successfully used to separate 14–20 LMW-GS from each NIL, and also distinguished 16 *Glu-3* NILs, except for Aroona-*Glu-B3c* and *B3d* without the length polymorphisms among *B3-688-1* and *B3-688-2* (Table 1; Figure 2). These results indicated that the LMW-GS gene marker system was efficient and accurate not only in separating members of the LMW-GS gene family [21], but also in distinguishing allelic variants of individual LMW-GS genes. Recently, allele-specific markers were widely used in distinguishing *Glu-A3* and *Glu-B3* alleles [12-14]. However, the high conservation among *Glu-D3* allelic variants made it difficult to develop allele-specific markers for discriminating different allelic variants. Because these allelic variants showed DNA sequence polymorphisms in length, the LMW-GS gene marker system worked well in dissecting the complex genes and allelic variants at the *Glu-D3* locus (Table 1). On the other hand, compared with only one gene in individual alleles identified with gene-specific primers [13,14], almost all the genes in *Glu-3* alleles were displayed using the LMW-GS gene marker system (Table 1). Identification of all genes in each allele will greatly contribute to an understanding of the molecular mechanism determining functional differences among *Glu-3* alleles in bread wheat.

In the present study, Aroona and its NILs contained most of the *Glu-3* allelic variations identified from the worldwide wheat germplasm in a cooperative program [12]. Each LMW-GS allele was encoded by several linked genes or haplotypes [13,14,16,17,21,22]. The dissection of LMW-GS genes in Aroona NILs would facilitate the haplotype analysis of LMW-GS genes in common wheat. At the *Glu-A3* locus, besides two m-type genes, 2–4 i-type genes were identified from individual *Glu-A3* alleles. Unlike the conserved m-type genes, i-type genes were completely different among five alleles. These i-type genes might be tightly linked, forming unique haplotype in each *Glu-A3* allele, e.g. *A3-484*/*A3-565*/*A3-568*/*A3-662* in *Glu-A3d* (Table 1) [13,18,19,21]. At the *Glu-B3* locus, 1–2 m-type gene(s) and 1–3 s-type gene(s) were characterized in individual alleles. Generally, these s-type genes could be divided into two groups, one containing *B3-578*/*B3-544*/*593*/*601*/*607*/*B3-621*/*624*, the other having *B3-688*/*B3-691*/*813*/*N* (Table 1, Figure 2). These s-type genes in each group might cosegregate and form unique haplotypes in bread wheat [14]. Among four allelic variants of m-type gene *B3-530*, *B3-530-2* was present in both Aroona (*Glu-B3b*) and Aroona-*Glu-B3h*, containing difference s-type haplotypes *B3-578*/*B3-607*/*B3-621-1* and *B3-688*/*B3-813*, respectively. Thus, at the *Glu-B3* locus, the m- and s-type genes appeared not to be tightly linked (Table 1; Figure 2) [6,18]. All the *Glu-D3* alleles except *Glu-D3f* contained eight LMW-GS genes (Table 1). Only one or two allelic variants (*D3-432*/*441*, *D3-525*/*528* and *D3-578-1*/*-2* were identified for each LMW-GS gene and only a few SNPs were detected among allelic variants. Thus, *Glu-D3* genes were highly conserved among wheat varieties [16,17], wherease *Glu-A3* and *Glu-B3* genes showed high diversities, which were derived from multiple allelic variations of i- and s-type genes, respectively.

To analyze differences in seed storage proteins among LMW-GS NILs, flour proteins were displayed using 2D-PAGE (Figures 3, 4 and 5). LMW-GS proteins shared some similar characteristics, such as mostly located in the B-group region and larger pIs than gliadins. Although it is difficult to detect small variation in molecular weight and quantity of each protein spot in 2D-PAGE, the composition and allelic variations of seed storage proteins for each *Glu-3* allele were successfully characterized. These results were in agreement with the 2D protein patterns of Xiaoyan 54, Jing 411, Norin 61, Butte 86 and some other varieties identified with LC-MS or N-terminal sequencing [18,19,36]. For example, the *Glu-A3d* and *Glu-B3d* alleles showed similar electrophoretic patterns to Xiaoyan 54 and Norin 61 in i-type (A3-568 and A3-662) and s-type (B3-688) proteins, respectively [18,19], and *Glu-B3g* shared the same spot pattern of B3-544 with Glenlea [19]. Moreover, comparison of 2D-PAGE patterns showed that, except for the different LMW-GS proteins, only 1–3 unique small gliadin spots were present in individual Aroona NILs (Figures 3, 4 and 5). These results suggested that the differences among the Aroona NILs were mainly derived from allelic variation of LMW-GS genes.

## LMW-GS alleles and bread-making quality

The NIL population was used to study the effects of LMW-GS on dough properties, GMP parameters, and pan bread quality parameters. The results confirmed that the LMW-GS played important roles in determining variation in wheat quality properties. Among the three *Glu-3* loci, alleles *Glu-A3* and *Glu-B3* were of major importance in determining differences in processing qualities among the NILs (Table 2).

### *Glu-A3* alleles

Among five *Glu-A3* alleles, *Glu-A3e* (*A3-391*, *A3-400*, *A3-502-3* and *A3-646*) performed the poorest in almost all quality properties (Table 1, Figure 6; Additional file 2: Tables S2, S3 and S4). This was consistent with previous reports in which *Glu-A3e* was associated with lower extensibility and Rmax than *Glu-A3d*, *A3b* and *A3c*[33]. The negative effect of *Glu-A3e* on dough rheological properties was reported in several studies previously [24,33,37,38]. No unique i-type protein band was detected from the *Glu-A3e* allele using SDS-PAGE (Figure 1a) [39]. The protein product of the *A3-646* gene was also not identified in 2D-PAGE although the i-type gene *A3-646* in Aroona-*Glu-A3e* contained the intact ORF (Figure 3b, Additional file 1: Figure S2). Less i-type proteins and more gliadins in *Glu-A3e* increased the ratio of gliadin to glutenin and greatly reduced %UPP (Figure 6; Additional file 2: Table S4), resulting in the worst performance of the *Glu-A3e* genotype in dough resistance and extensibility and pan bread total score [39]. The other *Glu-A3* NILs produced similar quality parameters, including Rmax, ST, Ext and %UPP. These data indicated that *Glu-A3a*, *A3c*, *A3d* and *A3f* all had equivalent positive effects on UPP content and dough resistance and extensibility (Figure 6). Among them, *Glu-A3d* had a significant effect on high ZSV, which was consistent with results from Xiaoyan 54 and Jing 411 RILs [18]. Some other studies also reported that the *Glu-A3d* allele had a superior effect on dough strength [40-42]. Compared with only one active gene at the *Glu-A3* locus in the other alleles, the *Glu-A3d* allele possessed three active LMW-GS genes and produced the highest ZSV, Rmax, and %UPP (Figure 6). The large number of active genes in *Glu-A3d* might be the basis of the superior performance in wheat quality properties [18].

### *Glu-B3* alleles

Our study confirmed the important contribution of the *Glu-B3* locus to quality variation of bread wheat varieties. Among eight *Glu-B3* alleles, *Glu-B3c* (*B3-530-1*, *B3-548* and *B3-688-1*) produced the lowest quality parameters, including ZSV, ST, Rmax, %UPP, and PBTS (Figure 6). The inferior effects of *Glu-B3c* allele were also reported in previous studies [24,33,43,44]. *Glu-B3c* and *Glu-B3d* (*B3-530-1*, *B3-548* and *B3-688-2*) shared an identical LMW-GS composition, except for five amino acid differences between B3-688-1 and B3-688-2 (Table 1; Figure 2, Additional file 1: Figure S5), but produced significantly different Rmax and ZSV (Figure 6). *B3-688-2* appeared to produce better quality properties than *B3-688-1*. On the other hand, *Glu-B3c* and *Glu-B3h* (*B3-530-2*, *B3-548, B3-688-3* and *B3-813*) also contained similar active LMW-GS genes and had only a few SNPs in the *B3-530* and *B3-688* allelic variants, but *Glu-B3h* had significantly more positive effects on PBTS and ZSV than *Glu-B3c* (Figure 6). The 2D-PAGE showed that *Glu-B3c* had a smaller quantity of B3-688-1 protein than *Glu-B3d* and *B3h* (Figure 4, Additional file 1: Figure S6). This suggested that the unique SNPs in *B3-688-1* might result in lower expression or difficulty in translation and polymerization. The small amount of B3-688-1 might directly cause the higher ratio of gliadin to glutenin and the lower %UPP for the *Glu-B3c* allele, finally resulting in its inferior effects on wheat quality (Additional file 2: Tables S2, S3 and S4). Compared to two active genes in the three *Glu-B3* alleles above, *Glu-B3i* contained four active genes, m-type haplotype *B3-510*/*B3-570* and s-type haplotype *B3-688*/*B3-691*. Both haplotypes provided the highest number of active genes and lead to the best quality performance among the four alleles [18].

The *Glu-B3a*, *B3b*, *B3f* and *B3g* alleles belonged to the same group of s-type haplotypes. They shared similar electrophoretic patterns and each possessed three active LMW-GS genes, but had significantly different effects on quality properties (Figure 6). *Glu-B3g* (*B3-530-3*, *B3-548*, *B3-544* and *B3-621-2*) produced the best quality parameters, including ZSV, Rmax, ST, %UPP, LV and PBTS (Figure 6), in agreement with previous reports [40,42,45]. *Glu-B3b* (*B3-530-2*, *B3-548*, *B3-607* and *B3-621-1*) also had positive effects on most quality parameters (Figure 6), again consistent with the previous studies [46,47]. The SE-HPLC parameters indicated that the low content of gliadin and the high content of UPP might be the main reasons for the superior performance of *Glu-B3b* and *B3g* (Figure 6; Additional file 2: Table S4). And the high content of UPP might be derived from the SNPs or InDels in the nucleotide sequences of three active genes (*B3-530-2*/*3*, *B3-544*/607 and *B3-621-1*/*-2*), which enhanced the ability of the protein products to form large glutenin macropolymers. More evidence was obtained by comparing *Glu-B3f* and *Glu-B3g*. Both alleles contained the same m-type gene, *B3-530-3*, but had different s-type haplotypes and significant differences in ZSV, ST, UPP, and PBTS. The s-type haplotype *B3-578*/*B3-544*/*B3-621-2* formed more UPP and produced better bread-making quality than *B3-578*/*B3-601*/*B3-621-1*.

### *Glu-D3* alleles

The five *Glu-D3* alleles produced similar values for almost all quality properties in the present study (Figure 6; Table 2), which confirmed previous reports that *Glu-D3* alleles produced similar Rmax and extensibilities among large collections of wheat varieties [24,33,48], although some studies indicated different effects of *Glu-D3* alleles on dough strength or mixing properties [27,29,38,49]. *Glu-D3a*, *D3b*, *D3c* and *D3d* contained similar 2D-PAGE spot patterns, and all six active genes were highly conserved among the alleles (> 99% identities). Their similarity in LMW-GS genes and whole proteins was consistent with their equivalent effects on all quality properties. Although *Glu-D3f* allele lacks two LMW-GS proteins and three gliadin spots, it produced similar quality properties to the other *Glu-D3* alleles (Figure 5; Table 2). These results suggested that *D3-385*, *D3-432* and the three missing gliadins were not related to quality improvement. However, the lack of active genes *D3-394* and *D3-528* at the *Glu-D3* locus in Jing 411 (*Glu-D3l*) showed significant negative effects on ZSV [18]. Thus, except for *Glu-D3l*, *Glu-D3* alleles appeared to play only minor roles in determining quality variation among bread wheat varieties, and they should be given the lowest priority among LMW-GS alleles in selecting for improved bread-making quality in wheat.

## Conclusion

In the present study, we dissected the genetic and protein composition of 16 LMW-GS NILs, measured the dough property and bread-making quality properties of individual NILs, and performed functional analyses for each allele. Among five *Glu-A3* alleles, *Glu-A3e* (i-type haplotype *A3-502-3*/*A3-646*) was inferior with negative effects on all quality properties. Among eight *Glu-B3* alleles, *Glu-B3b* (m-type gene *B3-530-2* and s-type haplotype *B3-578*/*B3-607*/*B3-621-1*), *Glu-B3g* (m-type gene *B3-530-3* and s-type haplotype *B3-578*/*B3-544*/*B3-621-2)* and *Glu-B3i* (m-type haplotype *B3-510*/*B3-570* and s-type haplotype *B3-688-4*/*B3-691*) were correlated with superior bread-making quality, whereas *Glu-B3c* (m-type gene *B3-530-1* and s-type haplotype *B3-688-1*/*N*) produced inferior quality properties. Among five *Glu-D3* alleles, there were no significant differences in all quality parameters measured in the present study. Moreover, all alleles with superior dough properties and pan bread quality also possessed high contents of UPP and %UPP. Thus, it is possible that LMW-GS alleles determine dough viscoelasticity by modifying the size distribution of glutenin polymers and aggregative properties of glutenins [50]. These results significantly enhance our understanding of the composition of LMW-GS, confirm the strong effects of LMW-GS on not only dough extensibility but dough strength, and provide useful information for quality improvement in bread wheat.

## Methods

### Plant materials

The wheat variety Aroona and 15 near isogenic lines (NILs) were kindly provided by Dr. Marie Appelbee and Prof. Ken Shepherd, SARDI Grain Quality Research Laboratory, South Australia. Each NIL contains a unique LMW-GS allele from a donor variety added to Aroona (Additional file 2: Table S5). They were planted at the Xinjiang Academy of Agri-Reclamation Sciences, Shihezi, and Xinjiang Academy of Agricultural Sciences, Urumqi, Xinjiang province, in randomized complete blocks with two replications during the 2010 cropping season.

### Analysis of LMW-GS genes

Genomic DNA of 16 Aroona NILs was extracted from young leaves of seedlings following Saghai-Maroof et al. [51]. The LMW-GS genes were separated by the LMW-GS gene molecular marker system [22]. With the help of the LMW-GS genes available from the Aroona NILs and some other wheat varieties [10,13,14,17,18], LMW-GS genes were characterized using Lasergene software (DNAStar; http://www.dnastar.com/), ClustalW2 (http://www.ebi.ac.uk/Tools/msa/clustalw2/), and MEGA 5 software [52].

### Isolation and separation of LMW-GS proteins

Glutenin extraction was performed according to the method described by Singh et al. [11]. These proteins were separated by SDS-PAGE using the method described by Sunbrook and Russell [53]. Whole seed proteins were isolated from wheat flour based on the SDS/Phenol method [54] with some modifications. Briefly, proteins in 0.12 g flour were precipitated with 10% TCA/acetone at −20°C overnight. After centrifuging at 20,000 g at 4°C for 15 min, the pellet was washed three times with 80% acetone, then dried at 50°C. Whole proteins were extracted with SDS/Phenol buffer (50% Tris-phenol pH 8.0, 30% sucrose, 2% SDS, 0.1 M Tris–HCl pH 8.0 and 2% DTT). The upper phenol phase was transferred into a new 2 mL tube. A fivefold volume of methanol containing 0.1 M ammonium acetate was added to the tube. The proteins were deposited at −20°C for 10 min or overnight. After centrifuging at 20,000 g for 5 min at 4°C, the pellet was washed once with 100% methanol and twice with 80% acetone before briefly drying in air. The proteins were dissolved in isoelectric focusing (IEF) sample extraction solution and used in 2D-PAGE analysis according to Dong et al. [18]. The images of SDS-PAGE were analyzed using NIH ImageJ software program (http://rsb.info.nih.gov/ij/).

### Quality testing and evaluation of pan bread

Measures of grain hardness, protein content, Zeleny sedimentation values, Farinograph and Extensograph parameters, and pan bread qualities were performed by methods reported in He et al. [55]. The glutenin macro-polymer compositions were measured following Zhang et al. [56].

### Statistical analysis

The SAS statistical package (SAS Institute, Cary, NC) was used for data analysis. All statistical analyses were based on averaged data from two locations.

## Abbreviations

2D-PAGE: Two-dimensional gel electrophoresis (IEF × SDS-PAGE); %UPP: UPP/(UPP + EPP) × 100; ANOVA: Analysis of variance; CIMMYT: International maize and wheat improvement center; cMs: Centimorgans; DT: Farinograph development time (min); DTT: Dithiothreitol; EA: Energy area (cm^2^); EC: External color; EPP: Extractable glutenin polymeric protein; Ext: Extensograph extensibility (mm); Gli/Glu: Ratio of gliadin to glutenin; GMP: Glutenin macro-polymers; HMW-GS: High-molecular-weight glutenin subunits; HPLC: High-performance liquid chromatography; IC: Inner color; IEF: Isoelectric focusing; kDa: Kilodalton; KP: Kernel protein (%, 14% m.b.); LMW-GS: Low-molecular-weight glutenin subunits; LV: Loaf volume (cm^3^); LVS: Loaf volume score; NIL: Near-isogenic lines; ORFs: Open reading frame; PBTS: Pan bread total score; PCR: Polymerase chain reaction; pI: Isoelectric points; RILs: Recombinant inbred lines; Rmax: Extensograph maximum resistance (B.U.); SDS: Sodium dodecyl sulfate; SDS-PAGE: Sodium dodecyl sulphate-polyacrylamide gel electrophoresis; Sha: Shape; Smo: Smoothness; Spr: Springiness; ST: Farinograph stability time (min); Str: Structure; TCA: Trichloroacetic acid; TF: Taste flavor; Tris–HCl: Tris (hydroxymethyl) aminomethane hydrochloride; UPP: Unextractable glutenin polymeric protein; Wab: Farinograph water absorption (%); ZSV: Zeleny sedimentation value (mL).

## Authors’ contributions

XZ carried out SDS-PAGE, 2D-PAGE and LMW-GS gene marker system analyses and drafted the manuscript. HJ measured glutenin macro-polymers, dough properties and pan bread quality parameters and performed statistical analysis. YZ participated in the measurement of processing quality parameters. DL participated in the identification of LMW-GS genes and proteins with SDS-PAGE, 2D-PAGE and LMW-GS gene marker system and revised the manuscript. GL participated in the design of the study and quality parameter analysis. XX participated in the design of the study and revised the manuscript. ZH conceived of the study and participated in its design, coordinated the various research groups, and revised the manuscript. AZ conceived of the study and participated in its design, and revised the manuscript. All authors read and approved the final manuscript.

## Financial competing interests

· In the past five years have you received reimbursements, fees, funding, or salary from an organization that may in any way gain or lose financially from the publication of this manuscript, either now or in the future? Is such an organization financing this manuscript (including the article-processing charge)? If so, please specify.

No.

· Do you hold any stocks or shares in an organization that may in any way gain or lose financially from the publication of this manuscript, either now or in the future? If so, please specify.

No.

· Do you hold or are you currently applying for any patents relating to the content of the manuscript? Have you received reimbursements, fees, funding, or salary from an organization that holds or has applied for patents relating to the content of the manuscript? If so, please specify.

No.

· Do you have any other financial competing interests? If so, please specify.

No.

## Non-financial competing interests

Are there any non-financial competing interests (political, personal, religious, ideological, academic, intellectual, commercial or any other) to declare in relation to this manuscript? If so, please specify.

No.

## Supplementary Material

Additional file 1: Figure S1 Quantitative gel densitometry measurements of B-group proteins in *Glu-B3* NILs as determined using NIL ImageJ software program. Data points were normalized with respect to the Aroona value. **Figure S2:** Sequence alignments of i-type proteins identified in Aroona NILs. **Figure S3:** Sequence alignments of the B3-530 protein and its allelic variants identified in Aroona NILs. **Figure S4:** Sequence alignments of the B3-544/593/601/607 proteins identified in Aroona NILs. **Figure S5:** Sequence alignments of the B3-621/624 and B3-688 proteins identified in Aroona NILs. **Figure S6:** The differential display of B3-688 spots among Aroona-*Glu-B3c*, *B3d* and *B3h*. Spot volume values are expressed in percentages (%vol) of the total proteome. Click here for file

Additional file 2: Table S1 F values of one way ANOVA of wheat quality properties by locations and genotypes. **Table S2:** Comparison of pan bread quality properties among Aroona NILs. **Table S3:** Comparison of dough properties among Aroona NILs. **Table S4:** Comparison of glutenin macro-polymer properties among Aroona NILs. **Table S5:** Composition of glutenin alleles in Aroona and its NILs. Click here for file
