# Supplementary material for: Composition and functional analysis of low-molecular-weight glutenin alleles with Aroona near-isogenic lines of bread wheat
Source: BMC Plant Biol. 2012 Dec 22;12:243. doi: 10.1186/1471-2229-12-243 (PMC3562532; doi:10.1186/1471-2229-12-243)
Supplement: Additional file 1: Figure S1 — Quantitative gel densitometry measurements of B-group proteins in Glu-B3 NILs as determined using NIL ImageJ software program. Data points were normalized with respect to the Aroona value. Figure S2: Sequence alignments of i-type proteins identified in Aroona NILs. Figure S3: Sequence alignments of the B3-530 protein and its allelic variants identified in Aroona NILs. Figure S4: Sequence alignments of the B3-544/593/601/607 proteins identified in Aroona NILs. Figure S5: Sequence alignments of the B3-621/624 and B3-688 proteins identified in Aroona NILs. Figure S6: The differential display of B3-688 spots among Aroona-Glu-B3c, B3d and B3h. Spot volume values are expressed in percentages (%vol) of the total proteome. [file 1471-2229-12-243-S1.pdf]

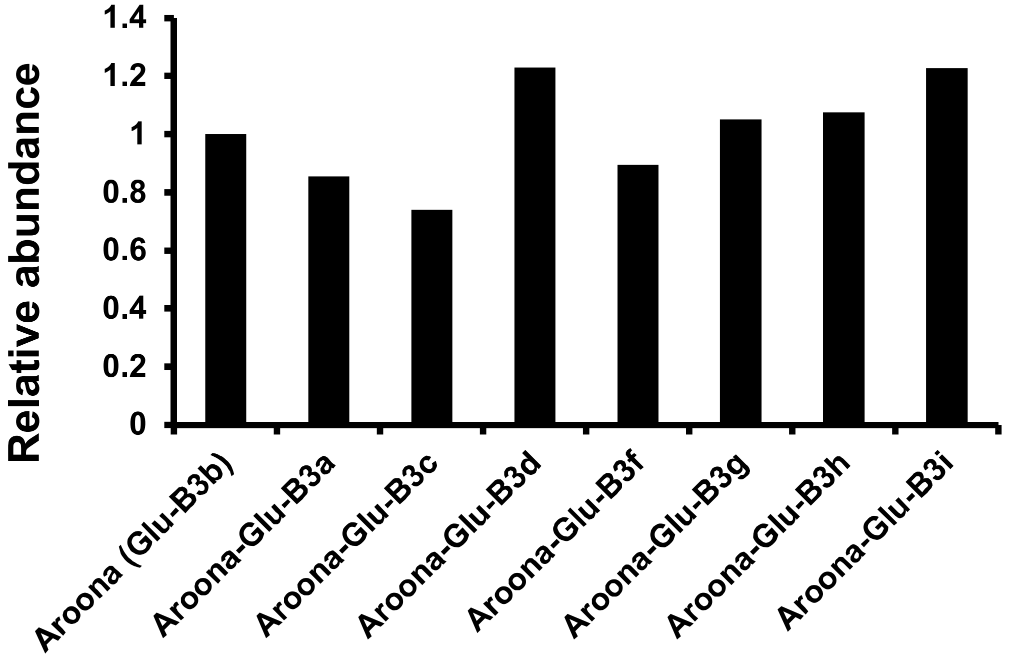

Supplementary Fig. 1 Quantitative gel densitometry measurements of B-group proteins in *Glu-B3* NILs as determined using NIL ImageJ software program. Data points were normalized with respect to the Aroona value.

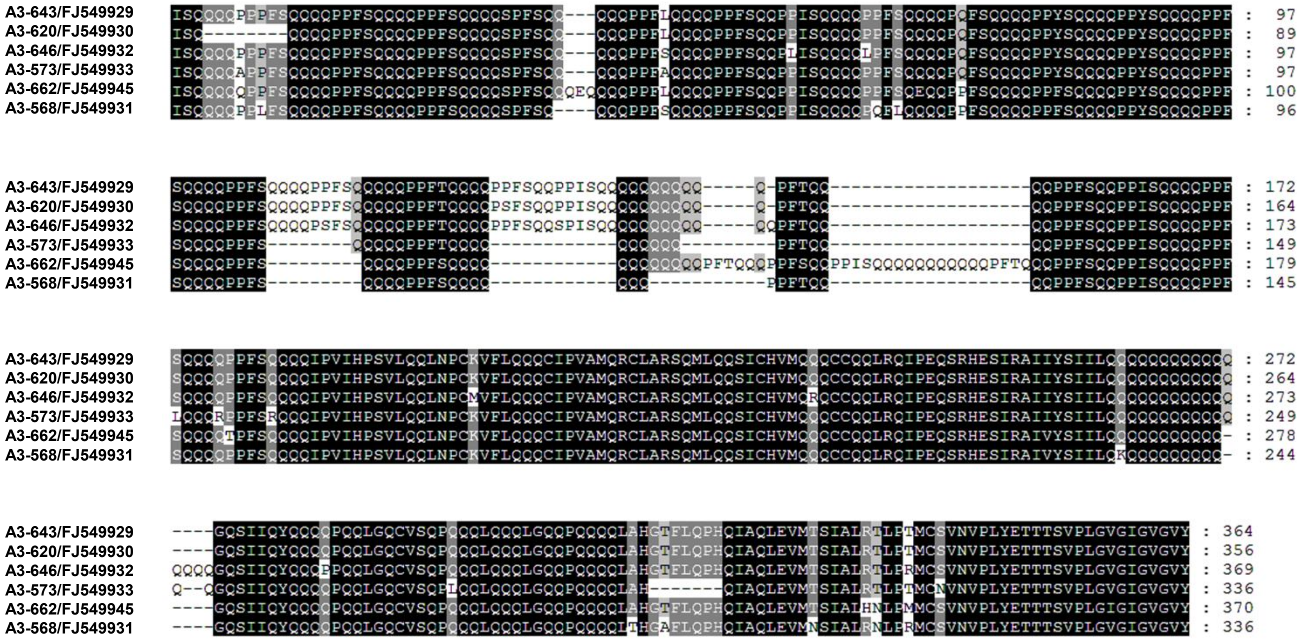

Supplementary Fig. 2 Sequence alignments of i-type proteins identified in Aroona NILs.

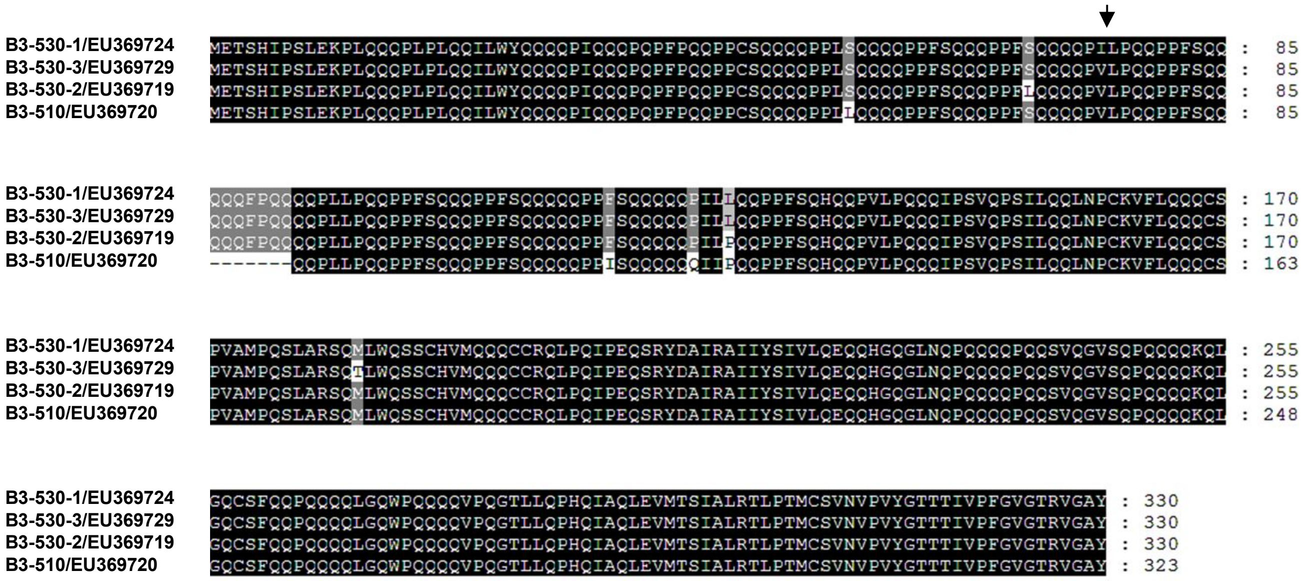

Supplementary Fig. 3 Sequence alignments of the B3-530 protein and its allelic variants identified in Aroona NILs.

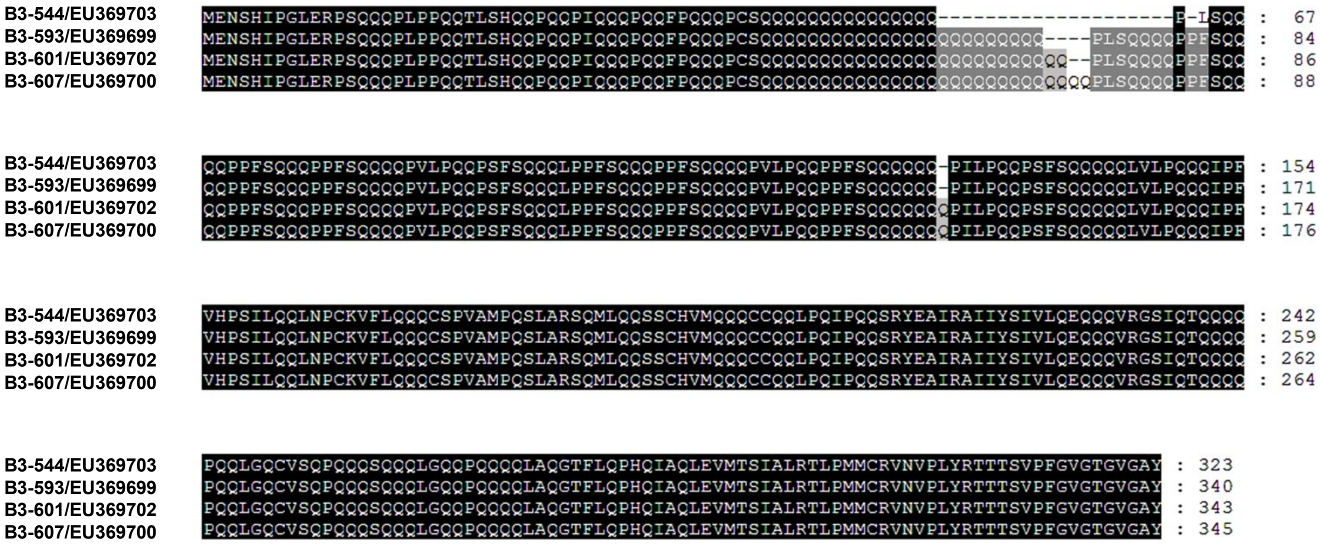

Supplementary Fig. 4 Sequence alignments of the B3-544/593/601/607 proteins identified in Aroona NILs.

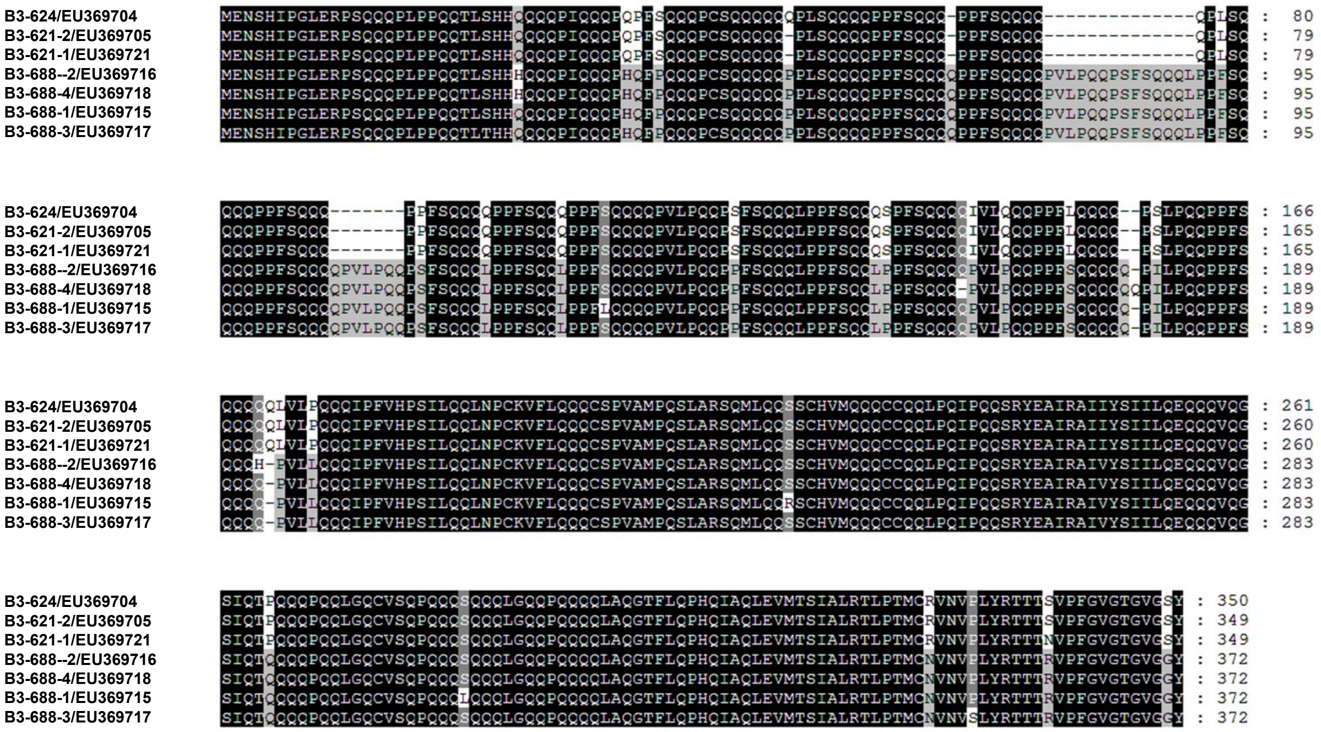

Supplementary Fig. 5 Sequence alignments of the B3-621/624 and B3-688 proteins identified in Aroona NILs.

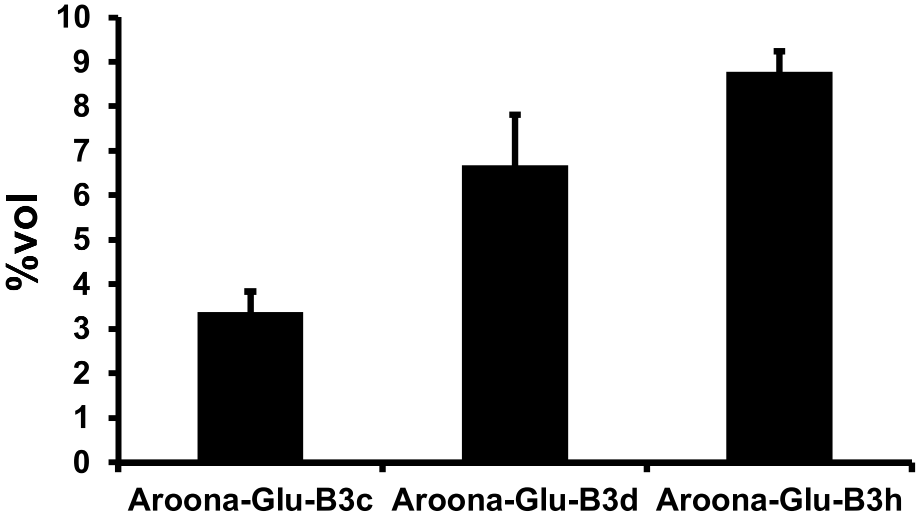

Supplementary Fig. 6 The differential display of B3-688 spots among Aroona-*Glu-B3c*, *B3d* and *B3h*. Spot volume values are expressed in percentages (%vol) of the total proteome
