# Supplementary material for: Composition and functional analysis of low-molecular-weight glutenin alleles with Aroona near-isogenic lines of bread wheat
Source: BMC Plant Biol. 2012 Dec 22;12:243. doi: 10.1186/1471-2229-12-243 (PMC3562532; doi:10.1186/1471-2229-12-243)
Supplement: Additional file 2: Table S1 — F values of one way ANOVA of wheat quality properties by locations and genotypes. Table S2: Comparison of pan bread quality properties among Aroona NILs. Table S3: Comparison of dough properties among Aroona NILs. Table S4: Comparison of glutenin macro-polymer properties among Aroona NILs. Table S5: Composition of glutenin alleles in Aroona and its NILs. [file 1471-2229-12-243-S2.pdf]

Supplementary Table 1: F values of one way ANOVA of wheat quality properties by locations and genotypes [a]

Source of variation	DF	LV	LVS	EC	Sha	IC	Smo	Spr	Str	TF	PBTS
Locations (L)	1	2.1	2.0	108.8**	8.2**	0.7	2.4	1.4	6.3*	13.4**	4.4*
Genotypes (G)	15	5.7**	3.9**	0.8	1.7	1.5	3.2**	0.7	2.8**	1.2	4.4**
L×G	15	1.0	0.4	2.4*	1.6	1.0	1.9	0.5	1.1	1.0	0.7

Source of variation	DF	KP	ZSV	ST	DT	Wab	Rmax	EA	Ext
Locations (L)	1	179.2**	370**	4.3*	1.0	23.1**	0.3	0.3	3.1
Genotypes (G)	15	3.2**	34.5**	6.8**	14.4**	6.4**	10.7**	10.0**	1.9
L×G	15	0.92	1.4	1.4	2.9**	1.3	1.9	1.3	0.6

source of variation	DF	Glutenin	Gliadin	Gli/Glu	EPP	UPP	%UPP
Locations (L)	1	22.5**	43.6**	0.6	15.3**	2.3	0.3
Genotypes (G)	15	1.2	4.4**	4.8**	2.2*	2.5*	3.5**
L×G	15	0.9	0.5	0.7	1.6	0.9	1.1

* Significant at *P*<0.05; ** Significant *P*<0.01.

LV: Loaf volume (cm^3), LVS: Loaf volume score, EC: External color, Sha: Shape, IC: Inner color, Smo: Smoothness, Spr: Springiness, Str: Structure, TF: Taste flavor, PBTS: Pan bread total score, KP: Kernel protein (%, 14% m.b.), ZSV: Zeleny sedimentation value (mL), ST: Farinograph stability time (min), DT: Farinograph development time (min), Wab: Farinograph water absorption (%), Rmax: Extensograph maximum resistance (B.U.), EA: Energy area (cm^2), Ext: Extensograph extensibility (mm), EPP: Extractable glutenin polymeric protein; UPP: Unextractable glutenin polymeric protein; Gli/Glu: Ratio of gliadin to glutenin; %UPP=UPP/(UPP+EPP) ×100.

Supplementary Table 2: Comparison of pan bread quality properties among Aroona NILs[a]

NIL	LV (cm^3)	LVS	EC	Sha	IC	Smo	Spr	Str	TF	PBTS
Aroona-*Glu*-A3b	719.8a	25.5a	4.5a	3.3a	3.1a	7.1b	8.0a	20.5a	3.9a	75.9ab
Aroona (*Glu*-A3c)	719.0ab	25.5a	4.4a	3.6a	3.3a	7.3b	8.4a	21.5a	4.1a	78.0a
Aroona-*Glu*-A3d	695.3bc	24.0a	4.5a	3.0a	3.0a	7.0b	7.6a	19.8a	3.9a	72.8bc
Aroona-*Glu*-A3e	682.3c	23.5a	4.6a	2.8a	3.0a	7.0b	7.9a	17.8b	4.0a	70.5c
Aroona-*Glu*-A3f	698.3abc	24.0a	4.4a	3.1a	3.0a	7.9a	8.1a	19.8a	4.1a	74.4abc
Aroona-*Glu*-B3a	708.8bc	25.0a	4.4a	3.3a	3.3a	7.3a	8.3a	19.5de	3.9a	74.8cde
Aroona (*Glu*-B3b)	719.0abc	25.5a	4.4a	3.6a	3.3a	7.3a	8.4a	21.5ab	4.1a	78.0abc
Aroona-*Glu*-B3c	715.3abc	25.0a	4.4a	3.0a	3.0a	6.6a	7.8a	18.8e	3.6a	72.1e
Aroona-*Glu*-B3d	700.8c	24.5a	4.3a	3.3a	3.0a	7.0a	7.8a	20.0cde	3.9a	73.6de
Aroona-*Glu*-B3f	709.5bc	24.5a	4.5a	3.1a	3.1a	7.1a	8.4a	20.5abcd	4.1a	75.4bcde
Aroona-*Glu*-B3g	740.8a	27.0a	4.3a	3.4a	3.3a	7.1a	8.5a	21.8a	4.1a	79.4a
Aroona-*Glu*-B3h	721.5abc	25.5a	4.4a	3.5a	3.3a	7.6a	8.3a	20.3bcd	4.1a	76.9abcd
Aroona-*Glu*-B3i	734.8ab	27.0a	4.5a	3.3a	3.0a	7.3a	8.1a	21.0abc	4.1a	78.3ab
Aroona-*Glu*-D3a	739.0a	27.0a	4.4a	3.3a	3.1a	7.6a	8.5a	20.8a	4.1a	78.8a
Aroona-*Glu*-D3b	726.0a	26.5a	4.3a	3.3a	3.1a	7.5a	8.4a	20.3a	4.0a	77.3a
Aroona (*Glu*-D3c)	719.0a	25.5a	4.4a	3.6a	3.3a	7.3a	8.4a	21.5a	4.1a	78.0a
Aroona-*Glu*-D3d	720.0a	25.5a	4.4a	3.3a	3.1a	8.1a	8.1a	20.0a	4.1a	76.6a
Aroona-*Glu*-D3f	747.3a	27.5a	4.6a	3.3a	3.5a	7.9a	8.3a	21.0a	4.1a	80.1a

[a] Statistical analysis of the quality parameters of alleles for each glutenin locus with ANOVA and Duncan LSR ($P<0.05$). The parameters are labeled by different letters or letter combinations based on multiple statistical comparisons. No statistical significance exists between the alleles labeled by one or more identical letters. LV: Loaf volume (cm^3), LVS: Loaf volume score, EC: External color, Sha: Shape, IC: Inner color, Smo: Smoothness, Spr: Springiness, Str: Structure, TF: Taste flavor, PBTS: Pan bread total score.

Supplementary Table 3: Comparison of dough properties among Aroona NILs[a]

NIL	KP (%)	ZSV (mL)	ST (min)	DT (min)	Wab (%)	Rmax (B.U.)	EA (cm^2)	Ext (mm)
Aroona-*Glu-A3b*	12.1a	46.4b	6.5a	4.8ab	62.8b	140.4a	41.5b	202.1ab
Aroona (*Glu-A3c*)	11.9a	44.3c	5.4a	4.6b	61.6c	156.8a	47.5ab	214.3a
Aroona-*Glu-A3d*	12.4a	48.7a	6.1a	5.0ab	60.7d	172.7a	51.3a	206.3a
Aroona-*Glu-A3e*	12.3a	38.3d	3.5b	3.8c	63.9a	90.0b	25.5c	188.5b
Aroona-*Glu-A3f*	12.1a	45.5bc	6.9a	5.3a	62.4b	166.8a	48.3ab	203.5a
Aroona-*Glu-B3a*	12.4a	42.9b	4.2cd	3.9cd	63.0ab	95.8cde	30.4bc	214.1a
Aroona (*Glu-B3b*)	11.9a	44.3b	5.4ab	4.6ab	61.6cd	156.8ab	47.5a	214.3a
Aroona-*Glu-B3c*	12.2a	35.9d	3.8d	3.6d	61.9bcd	66.3e	18.8d	189.2a
Aroona-*Glu-B3d*	12.0a	42.7b	4.5bcd	4.2bc	61.0d	107.7cd	31.0bc	182.2a
Aroona-*Glu-B3f*	12.1a	40.5c	4.3cd	4.3bc	62.9abc	130.9abc	40.7ab	212.0a
Aroona-*Glu-B3g*	12.1a	47.5a	6.1a	4.8a	62.6abc	164.4a	49.7a	211.7a
Aroona-*Glu-B3h*	12.1a	42.7b	4.6bcd	3.8cd	63.4a	84.6de	25.7cd	201.0a
Aroona-*Glu-B3i*	12.2a	44.3b	4.8bc	4.0c	60.9d	127.3bc	43.1a	226.2a
Aroona-*Glu-D3a*	12.1a	43.7a	5.6a	4.8a	61.4b	146.4a	45.2a	213.3a
Aroona-*Glu-D3b*	12.3a	43.5a	5.5a	4.8a	61.9ab	133.0a	41.6a	212.5a
Aroona (*Glu-D3c*)	11.9a	44.3a	5.4a	4.6a	61.6b	156.8a	47.5a	214.3a
Aroona-*Glu-D3d*	12.0a	45.0a	6.9a	5.2a	62.6a	166.1a	47.3a	200.1a
Aroona-*Glu-D3f*	12.3a	42.8a	5.9a	5.2a	62.2ab	163.6a	44.8a	195.3a

[a] Statistical analysis of the quality parameters of alleles for each glutenin locus with ANOVA and Duncan LSR ($P<0.05$). The parameters are labeled by different letters or letter combinations based on multiple statistical comparisons. No statistical significance exists between the alleles labeled by one or more identical letters.

KP: Kernel protein (%, 14% m.b.), ZSV: Zeleny sedimentation value (mL), ST: Farinograph stability time (min), DT: Farinograph development time (min), Wab: Farinograph water absorption (%), Rmax: Extensograph maximum resistance (B.U.), EA: Energy area (cm^2), Ext: Extensograph extensibility (mm).

Supplementary Table 4: Comparison of glutenin macro-polymer properties among Aroona NILs [a]

NIL	Glutenin (AU)	Gliadin (AU)	Gli/Glu	EPP (AU)	UPP (AU)	%UPP (%)
Aroona-*Glu-A3b*	16.9a	14.6a	0.87b	10.1a	5.7a	36.0a
Aroona (*Glu-A3c*)	16.6a	14.9a	0.90b	10.6a	5.9a	35.7a
Aroona-*Glu-A3d*	17.0a	15.5a	0.91b	10.3a	6.7a	39.4a
Aroona-*Glu-A3e*	16.2a	16.9a	1.05a	11.1a	4.6b	29.1b
Aroona-*Glu-A3f*	16.5a	15.3a	0.93b	10.0a	5.9a	37.0a
Aroona-*Glu-B3a*	16.9a	16.8a	1.00ab	11.6a	5.4bc	31.7bc
Aroona (*Glu-B3b*)	16.6a	14.9d	0.90d	10.6a	5.9ab	35.7ab
Aroona-*Glu-B3c*	16.2a	16.4ab	1.02a	11.3a	4.8c	29.9c
Aroona-*Glu-B3d*	16.7a	15.3cd	0.92bcd	10.6a	5.3bc	33.2bc
Aroona-*Glu-B3f*	16.3a	15.4cd	0.94abcd	10.4a	5.5bc	34.6ab
Aroona-*Glu-B3g*	16.1a	13.9e	0.87d	10.5a	6.4a	38.0a
Aroona-*Glu-B3h*	16.5a	16.3abc	0.99abc	11.0a	5.5bc	33.4bc
Aroona-*Glu-B3i*	17.1a	15.5bcd	0.91cd	10.9a	5.7ab	34.5ab
Aroona-*Glu-D3a*	16.4a	15.4a	0.94b	11.0a	6.2a	36.3a
Aroona-*Glu-D3b*	16.5a	15.2a	0.93b	9.7a	5.1a	32.9a
Aroona (*Glu-D3c*)	16.6a	14.9a	0.90b	10.6a	5.9a	35.7a
Aroona-*Glu-D3d*	16.3a	15.2a	0.93b	9.8a	6.8a	40.4a
Aroona-*Glu-D3f*	14.8a	15.3a	1.03a	9.9a	6.7a	40.5a

[a] Statistical analysis of the quality parameters of alleles for each glutenin locus with ANOVA and Duncan LSR ($P<0.05$). The parameters are labeled by different letters or letter combinations based on multiple statistical comparisons. No statistical significance exists between the alleles labeled by one or more identical letters.

EPP: Extractable glutenin polymeric protein; UPP: Unextractable glutenin polymeric protein; Gli/Glu: Ratio of gliadin to glutenin; %UPP=UPP/(UPP+EPP) ×100.

Supplementary Table 5: Composition of glutenin alleles in Aroona and its NILs

NIL	*Glu-A1*	*Glu-B1*	*Glu-D1*	*Glu-A3*	*Glu-B3*	*Glu-D3*	Donor Parent
Aroona	a	c	a	c	b	c	Aroona
Aroona-*Glu-A3b*	a	c	a	b	b	c	Gabo
Aroona-*Glu-A3d*	a	c	a	d	b	c	Orca
Aroona-*Glu-A3e*	a	c	a	e	b	c	Lerma Rojo
Aroona-*Glu-A3f*	a	c	a	f	b	c	Bungulla
Aroona-*Glu-B3a*	a	c	a	c	a	c	Chinese Spring
Aroona-*Glu-B3c*	a	c	a	c	c	c	Halberd
Aroona-*Glu-B3d*	a	c	a	c	d	c	Orca
Aroona-*Glu-B3f*	a	c	a	c	f	c	Gawain
Aroona-*Glu-B3g*	a	c	a	c	g	c	Millewa
Aroona-*Glu-B3h*	a	c	a	c	h	c	Sonalika
Aroona-*Glu-B3i*	a	c	a	c	i	c	Jufy 1
Aroona-*Glu-D3a*	a	c	a	c	b	a	Chinese Spring
Aroona-*Glu-D3b*	a	c	a	c	b	b	Bungulla
Aroona-*Glu-D3d*	a	c	a	c	b	d	Jufy 1
Aroona-*Glu-D3f*	a	c	a	c	b	f	India 115
